# Protease Activated Receptor 1 and Its Ligands as Main Regulators of the Regeneration of Peripheral Nerves

**DOI:** 10.3390/biom11111668

**Published:** 2021-11-10

**Authors:** Elena Pompili, Valerio De Franchis, Claudia Giampietri, Stefano Leone, Elena De Santis, Francesco Fornai, Lorenzo Fumagalli, Cinzia Fabrizi

**Affiliations:** 1 Department of Anatomy, Histology, Forensic Medicine and Orthopedics, Sapienza University of Rome, Via A. Borelli 50, 00161 Rome, Italy; defranchis.1450232@studenti.uniroma1.it (V.D.F.); claudia.giampietri@uniroma1.it (C.G.); elena.desantis@uniroma1.it (E.D.S.); lorenzo.fumagalli@fondazione.uniroma1.it (L.F.); cinzia.fabrizi@uniroma1.it (C.F.); 2 Department of Science, Roma Tre University, Viale Guglielmo Marconi 446, 00146 Rome, Italy; Stefano.leone@uniroma3.it; 3 Department of Translational Research and New Technologies in Medicine and Surgery, University of Pisa, Via Roma 55, 56126 Pisa, Italy; francesco.fornai@med.unipi.it; 4 I.R.C.C.S. Neuromed, Via Atinense 18, 86077 Pozzilli, Italy

**Keywords:** thrombin, protease-activated receptor, PAR1, peripheral nerve, regeneration, Schwann cells

## Abstract

In contrast with the brain and spinal cord, peripheral nerves possess a striking ability to regenerate after damage. This characteristic of the peripheral nervous system is mainly due to a specific population of glial cells, the Schwann cells. Schwann cells promptly activate after nerve injury, dedifferentiate assuming a repair phenotype, and assist axon regrowth. In general, tissue injury determines the release of a variety of proteases which, in parallel with the degradation of their specific targets, also activate plasma membrane receptors known as protease-activated receptors (PARs). PAR1, the prototypical member of the PAR family, is also known as thrombin receptor and is present at the Schwann cell plasma membrane. This receptor is emerging as a possible regulator of the pro-regenerative capacity of Schwann cells. Here, we summarize the most recent literature data describing the possible contribution of PAR1 and PAR1-activating proteases in regulating the regeneration of peripheral nerves.

## 1. Introduction

Diseases affecting the peripheral nervous system (PNS) are common and they often result in an impairment of sensory and/or motor conduction [1]. Stretch, lacerations, and compression of peripheral nerves may lead to disabilities in motor, sensory, and autonomic functions [2]. Paralysis of affected limbs and progression of neuropathic pain are common symptoms generated by deficiencies in motor and sensory functions [3]. Although peripheral nerves own a striking regenerative potential, regeneration can be limited by several factors such as age or area of the lesion. When nerve injuries are severe, they have destructive impacts on the life quality of patients. Neurons undergo degenerative changes through damage-induced interruption of the flow of neurotrophic factors from periphery to neuronal body by retrograde transport [4]. In the less severe nerve injuries mainly due to compression, the damage is restricted to the myelin sheath and the loss of function is only temporary. On the other hand, when the integrity of the axon is compromised, Wallerian degeneration occurs in the portion of the nerve distal to the lesion. In this instance, axonal regeneration is eased if the connective tissue surrounding the nerve fibers maintains its integrity. Conversely, in the case of complete nerve transection, the tentative regrowth of the axon often causes the formation of neuromas and rarely determines the successful reinnervation of the target tissue.

In peripheral nerves axons are surrounded by Schwann cells (SCs), a population of glial cells which provide metabolic and trophic support. Large diameter axons, which are a few sensory axons and axons from motor neurons, are wrapped by myelinating SCs. Non-myelinating SCs, also known as Remak cells, envelope small axons including many sensory axons and axons of the autonomic nervous system to form Remak bundles [5]. In rat sciatic nerve, the Remak bundles mostly comprise C-fibers nociceptors (about 48% of total axons) and postganglionic sympathetic fibers (about 23% of total axons) [6].

After nerve injury both Remak and myelin SCs convert to a phenotype known as “repair SC”, which is specialized in encouraging regeneration. Unfortunately, in humans, regeneration of peripheral nerves is often poor. One of the major problems is that the repair SC phenotype is unstable and fades with time in the chronically denervated distal stump. In addition, the pro-survival activity of repair SCs tends to decline with aging. The reason for the deterioration of repair cells is poorly understood and the specific pathogenesis of the conduction disorder of peripheral nerves is not completely clear. Possible exacerbation factors in peripheral nerve injuries may include excessive inflammation and coagulation. Of note, at the site of nerve injury a myriad of proteases is released from the damaged tissue. In particular, thrombin levels rise significantly over 24 h following peripheral nerve injury [7].

Thrombin is well-known as a key mediator of the coagulation cascade. However, it is also an important regulator of the inflammatory responses. The plasma concentration of prothrombin in normal subjects is between 700 nM and 1.7 µM [8]. Importantly, thrombin and FX/FXa can also be generated locally in peripheral nerves [9]. Low concentrations of thrombin have been demonstrated to improve the regeneration of mouse peripheral nerve after injury [10]. Conversely, thrombin at high concentrations worsened the degeneration of crushed nerves [11]. The main thrombin receptor is the protease-activated receptor 1 (PAR1) that is found at the intersection of two pathways, coagulation and inflammation. PAR1 is expressed by SCs, especially at the level of the microvilli in the region of the nodes of Ranvier [12,13].

Here, we review the most recent findings highlighting the possible role of PAR1 and thrombin in regulating peripheral nerve regeneration and considering the contribution of other potential PAR1-activating proteases. We performed a literature search on PubMed for articles published between 1 January 1979 and 1 September 2021. The search terms were “Schwann cell” OR “peripheral nerve” AND the following proteases: “Thrombin”, “Factor X” OR “FX”; “Factor VII” OR “FVII”; “activated protein C” OR “APC” OR “Endothelial Protein C Receptor” OR “EPCR”; “plasmin” OR “tissue plasminogen activator” OR “tPA” OR “urokinase plasminogen activator” OR “uPA”; “matrix metalloproteinase” OR “MMP”. With regards to the MMPs, we focused on the proteases that can cut and activate PAR1 (namely MMP2, MMP3, MMP8, and MMP9). We excluded comments, case reports, and articles which were not related to peripheral nerves.

## 2. Schwann Cells in the Physiology and Pathophysiology of Peripheral Nerves

Axonal regeneration after peripheral nerve injury is potently guided and supported by Schwann cells (SCs), a population of glial cells ensheathing the axons in the PNS [14,15]. By wrapping the axon with many layers of membranes, SCs form the myelin sheath of the PNS of vertebrates [16]. The insulation of the axon by myelin increases dramatically the conduction speed restricting action potentials to the nodes of Ranvier. Therefore, myelin by ensheathing the large diameter axons generates the saltatory impulse propagation and speeds up nerve conduction velocity by 20–100-fold [17,18]. Besides, in the myelinated fibers, a linear dependency occurs between the speed of nerve conduction and the axonal diameters [19,20]. In addition to their main function, which is the synthesis and maintenance of the myelin sheath, SCs strictly cooperate metabolically with the axonal compartment that may extend far away from the correspondent neuronal soma [15]. In particular, the axonal compartment faces a narrow subcompartment of the glial cell, which is the adaxonal myelin layer. In this region, the myelinated axon and the associated SC results metabolically coupled [21].

SCs derive from the neural crest and undergo three pivotal stages of development [22,23,24]. Initially, during the early embryonic development the migrating neural crest cells generate multipotent progenitors, the SC precursors. Next, during late embryonic development SC precursors differentiate into immature SCs. Then, the immature SCs go through numerous phenotypic changes, exit the cell cycle, and form the mature SCs that are present postnatally. Mature SCs are generally divided in two major subtypes: myelinating and non-myelinating (also known as Remak) SCs. Initially, immature SCs extend their lamellae and contact a bundle of unmyelinated axons. When the diameter of the axon is >1 µm, the SCs establish a 1:1 contact ratio and produce myelin sheaths [25,26] expressing characteristic myelin markers, such as protein 0 (P0), myelin-associated protein (MAG), and galactocerebroside (Gal-C) [27]. Conversely, when SCs contact small diameter axons, such as those of nociceptive neurons, they become non-myelinating SCs and maintain some markers typical of the immature states, such as the cell adhesion molecule L1 and the p75 neurotrophic receptor (p75NTR) [28].

SCs are enveloped by basal lamina and surrounded by the endoneurium formed by fibroblasts, blood vessels, and hardly any macrophages. The endoneurium is, in turn, enveloped by another layer of connective tissue called perineurium, forming a fascicle. Large nerves contain many fascicles that are held together by the epineurium.

Following nerve injury, the denervated SCs distal to the damaged area gain a repair phenotype that only partially mimics the immature SCs. When SCs get in contact with the degenerating axon, these cells re-enter the cell cycle and promptly start to proliferate. In so doing, repair SCs downregulate genes necessary for myelin maintenance and axon support (such as krox-20; myelin basic protein, MBP and periaxin), and conversely upregulate genes required for repair and regeneration (such as glial cell line-derived neurotrophic factor, GDNF; brain-derived neurotrophic factor, BDNF; neurotrophin-3, NT-3; nerve growth factor, NGF and p75NTR). Nerve injury triggers the strong activation of mielinophagy, a selective autophagic removal of the myelin sheath [29]. It is known that during the first 5–7 days after injury, SCs are responsible for the degradation of about 40–50% of myelin [30]. Macrophages accelerate the removal of myelin in the later stages of Wallerian degeneration. Repair SCs release cytokines (such as interleukin-6 and leukocytosis-inducing factor, LIF) recruiting macrophages and other immune cells to the site of injury. The rapid degradation of myelin and the removal of cell debris by the SCs through both phagocytosis and autophagy are fundamental to ensure and facilitate an efficient nerve regeneration [29,31]. Central in the reprogramming of SCs to a repair phenotype after injury is the transcription factor c-Jun [32,33] and pharmacological or genetic inhibition of JNK1/c-Jun reduces the SC autophagic flux [34]. Although referred to the central nervous system, it is worth mentioning that in hippocampal neurons thrombin signals through PAR1 to JNK transiently activating the transcription factor c-Jun with different kinetics for the high and low levels of the stimulus [35]. During nerve degeneration and regeneration processes, repair SCs undergo profound functional and morphological changes. In particular, repair SCs become 7- to 10-fold longer than immature SCs giving rise to the formation of the Büngner’s bands. The Büngner’s bands, formed by endoneurial tubes surrounding the repair SCs, guide and promote the extension of the regenerating axons. In the distal stump of the injured nerve, endoneurial fibroblasts and repair SCs together create a permissive environment which is able to support the re-growth of the damaged axon. The prominent ability of peripheral neurons to regenerate their axons after lesion is usually not sufficient to warrant an adequate functional recovery. In fact, a successful restitution requires that the appropriate axon reinnervates the target organ. In general, efficient functional recovery is well achieved by regenerative thin nerve fibers, such as fibers responsible for thermal and pain sensitivity and sympathetic efferents. Conversely, for the large nerve fibers innervating muscles and specialized mechanoreceptors, the functional recovery after injury is often poor with no clear distinction between motor and sensory fibers of the same caliber [36]. 

Unfortunately, in humans, regeneration of peripheral nerves is often poor as the phenotype of repair SCs fades with time and tends to decline with aging. In nerve crush injuries, the basal lamina remains intact and the axon can grow following this path to reconnect to its target. Conversely, when the nerve is cut, the continuity of both the basal lamina and the surrounding connective tissue is compromised and the regrowing axon is less likely to reconnect to its proper target. Regardless, if the nerve is damaged by a crush or cut, the SCs response to injury and the generation of repair SCs will always occur. There are currently no treatments to successfully foster myelin regeneration after injury of the PNS. Therefore, there is a great need to identify druggable target proteins that are potential modulators of PNS regeneration.

Recently, the protease-activated receptor 1 (PAR1) has been described by our group and others as a possible regulator of SC pro-regenerative potential [37,38,39,40].

## 3. Protease-Activated Receptor 1 (PAR1) General Features and Activation Mechanism

PAR1, together with the other three members of the PAR family (PAR2, PAR3, and PAR4), is a G protein-coupled receptor (GPCR) belonging to the large Rhodopsin family. PAR1 is activated by the cleavage of its N-terminus [41,42,43,44], which contains an hirudin-like domain with a high-affinity binding site for thrombin [41]. Moreover, PAR1 can be cleaved and activated by several other proteases and coagulation factors, including Factor Xa (FXa) [45,46], Factor VIIa (FVIIa) [47], plasmin [48], MMP2, MMP3, MMP8, MMP9 [49,50], trypsin [51], granzyme-A and B [52,53,54], and cathepsin-G [55]. Concerning the other PARs, PAR2 is activated by trypsin [42,56] and other proteases, such as tryptase [57], matriptase [58], FXa, FVIIa [58,59], plasmin [60], thrombin [61], and kallikrein (KLK)-5,-6,-14 [62]. PAR3 bears a reported cleavage site for thrombin but seems unable to generate any signal transduction [63]. PAR4 binds thrombin with low-affinity since, differently from PAR1, it lacks an hirudin-like domain [48,64]. In addition to thrombin [65], PAR4 can be also cleaved by trypsin [66] and cathepsin-G [67].

The PAR1 N-terminus is recognized and cleaved by thrombin at Arg41 (canonical R41-S42 site). This proteolytic cleavage generates a new N-terminus that corresponds to the “tethered ligand” and interacts with a conserved sequence in the extracellular loop 2 of the receptor. As mentioned above, the thrombin/PAR1 interaction is facilitated by the hirudin-like sequence of PAR1, which displays a high specificity and affinity for thrombin at exosite I. The presence of the high-affinity hirudin-like domain renders PAR1 an excellent substrate that can be activated by subnanomolar concentrations of thrombin [41]. The cleavage and consequent conformational change of PAR1 determines its coupling with multiple Gα proteins such as Gαq, Gα12/13, and Gαi [68]. When activated, PAR1 can enable the recruitment of β-arrestin and then the Rac1 and Akt activation [69]. The triggering of PAR1, which consists of a proteolytic cleavage of its N-terminus, is intrinsically irreversible. In fact, following cleavage, the newly generated N-terminus remains tethered to the receptor. Therefore, in order to interrupt PAR1 signaling, the ligand cannot merely diffuse away and the receptor itself needs to be desensitized and internalized. As observed for other GPCRs, PAR1 internalization by endocytosis requires both clathrin and dynamin [70,71]. Even when the stimulus is absent, PAR1 continues to circulate between the cell membrane and the intracellular compartment, thereby maintaining a stable pool of the receptor on the surface [72,73].

In general, PAR1 forms the link between coagulation and inflammation as it was reported to mediate the induction of both pro- and anti-inflammatory molecules [74,75]. For instance, in dendritic cells, the lethal inflammatory response sustained by the activation of PAR1 can be interrupted if thrombin or PAR1 signaling is suppressed [76]. Conversely, previous results obtained in microglia indicate that the activation of PAR1, but not PAR2, limits the production of lipopolysaccharide-induced pro-inflammatory mediators through the upregulation of the suppressor of cytokine signaling-3, SOCS-3 [77].

When PARs are proteolytically cleaved, a new N-terminal sequence indicated as “tethered ligand” is generated. The “tethered ligand” rapidly binds intramolecularly determining a conformational change of the receptor and thus, triggering a downstream intracellular signaling. PAR1, PAR2, and PAR4 are all coupled to Gαq/11, Gα12/13, and Gαi/o [68]. Moreover, it has been reported that PARs are able to activate certain signaling pathways through the interaction with β-arrestins [69].

The cleavage of PARs by thrombin and trypsin is commonly named canonical activation. Moreover, other proteases can cut the PAR N-terminus, but at distinct sites generating a novel “tethered ligand” by a noncanonical activation. In certain instances, the noncanonical proteolytic cleavage of PARs disarms the receptor by removing the activating “tethered ligand” and thus, by this event inhibiting PAR mediated signaling. In addition, PARs have been reported to transactivate or coactivate adjacent receptors by dimerization or sequestration in membrane microdomains. Transactivation occurs when the tethered ligand of a proteolytically activated PAR binds to an adjacent receptor. On the other hand, coactivation occurs when a PAR receptor binds a protease with high affinity and then, this protease cuts and activates an adjacent PAR. As an example, thrombin bound with high affinity to the hirudin-like domain of PAR1 and PAR3 can cut and activate PAR4 [78,79]. 

In general, biased agonism is the ability of different ligands to act at the same GPCR but triggering distinct cellular signaling (for a recent review, see [72,73]).

The role of PARs in regulating key processes such as coagulation, hemostasis, and inflammation are all well-recognized, and have been deeply investigated.

## 4. Protease-Activated Receptor 1 (PAR1)/Thrombin Axis in Peripheral Nerve Injury

**Figure 1:**
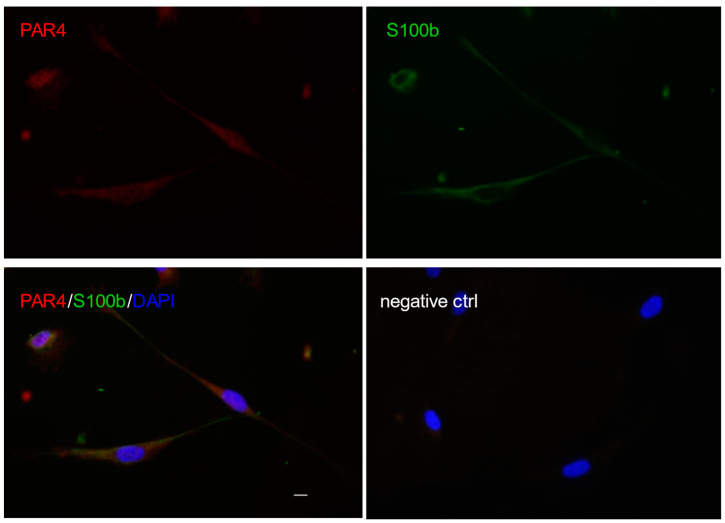
PAR4 detected by immunofluorescence in S100b positive Schwann cells from human spinal nerves (Sciencell). The negative control was performed by replacing the primary antibodies with equivalent amounts of normal Igs. Scale bar is 5 µm.

In the PNS, PAR1 is found at the SC plasma membrane and highly present on SC microvilli at the nodes of Ranvier [12,13]. PAR1 is the only PAR family member described to date in SCs [12,13,80], although our preliminary data indicate that PAR4 is also expressed by SCs at least in culture (Figure 1). Future studies aimed at identifying the presence and function of the four PAR family members in peripheral nerves are needed to clarify their role, if any, in PNS physiology and pathophysiology.

Numerous studies support the involvement of the thrombin pathway in SC mediated regeneration and axonal function. In models of peripheral nerve crush, the levels of both prothrombin and thrombin are found elevated [7,81]. In particular, an increased thrombin activity was measured both in the entire crushed nerve [7] and in the portion of the nerve distal to the lesion [81]. As mentioned above, low levels of thrombin enhance the regeneration of mouse peripheral nerve after crush injury [10], while high concentrations had detrimental effects [11]. Consistently, PAR1 activation with high concentrations of thrombin and PAR1 agonists causes a conduction block in motor nerve fibers [12]. Otherwise, low concentrations of thrombin display neuro-regenerative effects by inducing activated protein C (APC), which couples with its receptor, endothelial protein C receptor (EPCR), and activates PAR1 [82]. In this connection, it has been previously reported that PAR1 stimulation with low doses of thrombin and PAR1 agonist peptides increases neurotrophic and neuroprotective properties of cultured SCs [13,80]. Indeed, the activation of PAR1 by low levels of specific agonist peptides enhances the ability of SC cultures to release molecules already known to promote nerve regeneration such as decorin, macrophage migration inhibitory factor (MIF), and matrix metalloproteinase-2 (MMP2) [13]. Conversely, a reduced capacity of promoting the PC12 neurite extension with respect to the control is observed in cultured SCs treated with high levels of thrombin [80]. Despite the intrinsic limitations of these in vitro data, it is interesting to note that they resemble the results obtained in vivo with low [10] and high levels of thrombin [11]. Similar to the data obtained in SC cultures, a divergent effect of low and high levels of thrombin was observed in sciatic nerve ex vivo explants. This model system can partially resemble the distal portion of the nerve after transection where the axon is separated by the perikaryon, while the three-dimensional relationship between SCs and the axon is mainly maintained. In this model, low levels of thrombin are unable to determine any evident modification in the morphology of SCs and of the nodes. Conversely, increased concentrations of thrombin (or PAR1 agonist peptide) cause a profound rearrangement of SCs. In fact, high levels of thrombin in ex vivo nerve explants determine an evident paranodal demyelination, a dilation of the Schmidt-Lantermann incisures and the disappearance of the Cajal bands [80]. The Cajal bands, in particular, are cytoplasmic channels that facilitate the microtubule-based transport of proteins and organelles in SCs; aberrations in their architecture correspond to an impairment of myelin maintenance [83]. These data are consistent with the results obtained in CNS where PAR1 agonists, namely thrombin and PAR1-agonist peptides, mediate parallel oligodendrogliopathic effects [84]. In addition, the activation of PAR1 signaling has been demonstrated to improve the pro-regenerative capacities of cultured olfactory ensheating glia (OEGs), while the thrombin inhibitor trombomodulin negatively modulates axonal regrowth [37]. It is worth mentioning that OEGs are a population of glial cells associated with the small caliber axons of the olfactory neurons, that share with SCs the ability to promote axonal regeneration.

**Figure 2:**
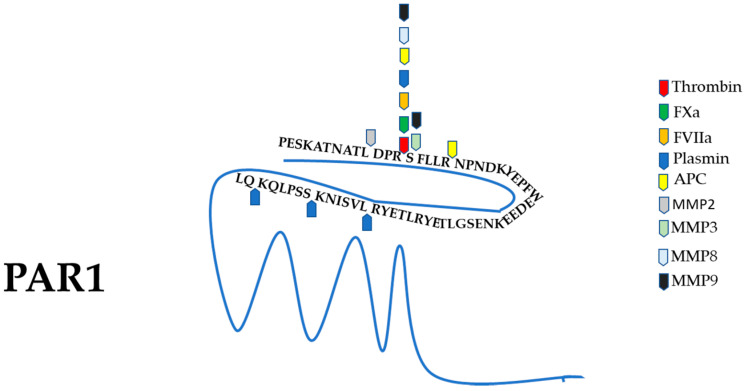
Schematic representation of canonical and noncanonical cleavage sites on the PAR1 N-terminus. PAR1 is canonically cleaved and activated by thrombin between R41 and S42. The same site is proteolitically cleaved by FXa, FVIIa, high levels of plasmin, and some MMPs. The cleavage of PAR1 at the noncanonical sites generally causes the disarming of the receptor. The canonical and noncanonical cleavage sites of the proteases reported in Table 1 are indicated.

The analysis of the possible role of PAR1 in regulating the regenerative processes in peripheral nerve injury gets further complicated by the possible involvement of other PAR1 agonists different from thrombin. It is now well known that numerous proteases can cleave PAR1 and that its activation depends on the membrane environment and also by the cofactors that are present. Besides, canonical and noncanonical activation of PAR1 may generate a different cellular response. Putative PAR1 activating proteases that have been reported to be possibly involved in peripheral nerve regeneration are briefly described below and summarized in Table 1. Their canonical and noncanonical cleavage sites on the PAR1 N-terminus are outlined in Figure 2.

**Figure 3:**
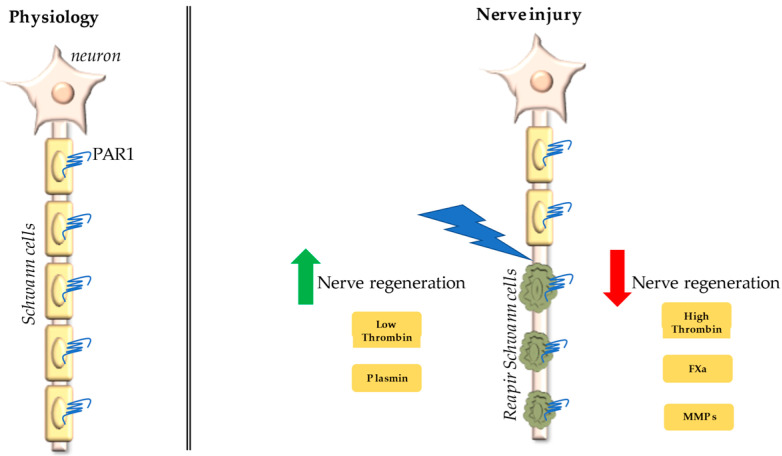
Proteases which have been reported to affect peripheral nerve regeneration after injury and that are also potential activators of PAR1.

Among the proteases reported in Table 1, some of them (such as low levels of thrombin and plasmin) have been shown to promote nerve regeneration, while many others (such as high levels of thrombin, FXa, and some MMPs) seem to inhibit nerve functional recovery after damage (Figure 3).

## 5. Activated Factor X (FXa) in Peripheral Nerve Injury

In addition to thrombin, another potent activator of PAR1 is FXa which drives platelet aggregation and thrombus formation [98].

In the blood, the activated Factor X (FXa) is derived from the cleavage of Factor X (FX) by the tissue factor (TF) and Factor VIIa (FVIIa), and it is responsible for the conversion of prothrombin into thrombin. Moreover, FXa can be generated through the intrinsic pathway by the activated FIX complexed with its cofactor activated FVIII. 

Interestingly, both FX and FXa are expressed in the sciatic nerve and appear localized in SCs at the level of the abaxonal membrane, which is the outer SC membrane in contact with the basal lamina. In addition, when the sciatic nerve gets injured initially the expression levels of both FX and FXa rise dramatically. At later times, the level of FXa declines slowly while its precursor FX remains highly expressed, indicating the possible interference of the conversion of FX to FXa by an endogenous inhibitor [9]. Although in these experiments blood vessels were removed from nerve samples, contaminations coming from residual small vessels cannot be excluded. Nevertheless, these data support the importance of FXa as the rate-determining step in the generation of thrombin and the activation of PAR1 during nerve damage. Of note, a specific FXa inhibitor restores the motor function after nerve crush injury in mice [9].

## 6. Activated Factor VII (FVIIa) in Peripheral Nerve

Factor VII (FVII) is one of the factors responsible for the generation of blood clots during coagulation. The activated Factor VIIa (FVIIa) is generated from FVII by the tissue factor (TF), which is released from the damaged tissues. Once generated, FVIIa then activates FIX and FX.

FVIIa is able to induce PAR1-dependent cytoprotective signaling through cleavage of PAR1 at the canonical site [99].

Both TF and FVII have been demonstrated to be present in PNS at the nodes of Ranvier with a localization similar to the one already reported for PAR1 [9].

## 7. Activated Protein C (APC) in Peripheral Nerve Injury

Protein C is activated by low levels of thrombin. In turn, activated protein C (APC), together with its receptor (endothelial protein C receptor, EPCR), cleaves and activates PAR1 [100]. EPCR is present at the level of SC microvilli and its expression markedly increases following injury in the distal segment of the crushed nerve [7].

APC is an anticoagulant enzyme triggering PAR1 but generating a distinct signaling response with respect to thrombin. In general, when activated by APC, the PAR1 limits proinflammatory signaling and protects cells from death [101]. APC induces PAR1 canonical proteolysis but also cleaves an alternative site between Arg46 and Asn47 (R46 N47) [102]. Differently from thrombin, the PAR1 activation by APC is observed in caveolar microdomains and appears to be mediated by β-arrestin recruitment and dishevelled-2 (Dvl-2) activation [103].

## 8. Plasmin in Peripheral Nerve Injury

Plasmin is the main enzyme in fibrinolysis. The tissue plasminogen activator (tPA) and urokinase (uPA) located on the surface of the fibrin clot or at the cell membrane generates plasmin from the zymogen plasminogen. tPA is predominantly present in the blood, although it is also found in other systems.

In PNS, following sciatic nerve injury, tPA is quickly induced in sensory neurons and SCs. In addition, the lack of tPA or plasminogen in rats worsens axonal degeneration and myelin sheath decomposition, while hindering the functional recoveries after sciatic nerve injury [104,105]. Consistently, Sajadi et al. [86] showed that the simple administration of tPA is able to favor the regeneration of damaged peripheral nerve. Although these previous data seem to indicate that the tPA/plasminogen system can contribute to the regulation of degeneration and regeneration of damaged peripheral nerves, there is not enough knowledge of how the exogenous tPA or tPA/plasminogen affects the nerve regeneration following an injury [85]. Plasmin is known to mediate PAR1 proteolysis at a noncanonical site. Therefore, it desensitizes the receptor to a possible further activation by thrombin. Moreover, when at high concentrations, plasmin can canonically activate PAR1 [48].

Furthermore, in the PNS, both the neurons and SCs express uPA, whose level increases after damage during the early stages of regeneration (1–7 days after injury) in peripheral nerves in vivo and in sensory neurons in vitro [87,105,106,107,108]. Therefore, both the tPA and uPA mediate an extracellular proteolytic cascade that seems to play a protective role against axonal degradation and demyelination, and to promote axonal regeneration after sciatic nerve crush [87].

## 9. Matrix Metalloproteinases (MMPs) in Peripheral Nerve Injury

Matrix metalloproteinases (MMPs) consist of a family of calcium-dependent zinc endopeptidases, which include soluble (collagenases, gelatinases, matrilysins, and stromelysins) and membrane-type MMPs [109]. Soluble MMPs are composed of an N-terminal inhibitory prodomain connected by a flexible linker region to an active site catalytic domain, and of a C-terminal hemopexin domain. The catalytically active protease is generated by the proteolytic removal of the inhibitory prodomain from the inactive proenzyme. MMP activation by proteolysis regulates a broad spectrum of biological events, ranging from the level of extracellular matrix components to the function of signaling receptors located at the plasma membrane [110]. The MMP activity can be hindered by four tissue inhibitors known as TIMPs, consisting of an N-terminal inhibitory and a C-terminal non-inhibitory domain [111].

In general, MMPs cleave PAR1 at noncanonical sites that are distinct from thrombin, thus generating a different tethered ligand [89]. The inhibition of MMP activity by a broad spectrum MMP inhibitor enhances the proliferation of cultured SCs in vitro and the rate of nerve regeneration in vivo [88]. MMPs that cleave PAR1 and that have a possible role in the regulation of the degeneration/regeneration processes of peripheral nerves are briefly described below. They comprehend MMP2, MMP3, MMP8, and MMP9.

As far as MMP2 is concerned, SCs have been identified as the main cellular source in peripheral nerves [112,113]. MMP2 has been described as displaying an important role in favoring axonal regeneration [114], particularly enhancing remyelination of regenerated axons [91]. MMP2 downregulation has been proposed to contribute to the deficits in nerve regeneration, which is observed in diabetes [115]. 

MMP3 is one of the major components of the inflammatory signaling pathway [116]. MMP3 has been observed to be produced and secreted by perisynaptic SCs [92]. Following nerve damage, the neuromuscular junctions are better preserved in MMP3 knockout mice than in controls [93].

MMP8 has been reported to be involved in the development of pruritus by regulating skin nerve density [94]. In particular, MMP8 guides sensory nerve growth within the interstitial collagen matrix by modulating axonal guidance molecules and extracellular matrix components.

Among gelatinases, MMP9 is involved in a variety of inflammatory, infectious, and repair processes, including sepsis, cancer, haemorrhage, and systemic autoimmunity [117]. The hemopexin domain of the MMP9 proenzyme binds the C-terminus of TIMP-1 forming stoichiometric (1:1) stable heterodimers, which are more resistant to activation with respect to the free MMP9 proenzymes.

In adult peripheral nerve, MMP9 is produced uniquely and shortly after damage. In particular, in the damaged adult peripheral nerve, the expression of MMP9 is induced in SCs and endothelial cells by proinflammatory cytokines, such as TNF-α and IL-1β [95,96]. Lately, infiltrating immune cells, including neutrophils and macrophages, serve as additional sources of MMP9 in the injured nerve. MMP9 plays multiple roles during the early phase of nerve injury including regulation of blood-nerve barrier breakdown, recruitment of immune cells, glial activation, demyelination/remyelination, and pain [90]. In sciatic nerve chronic constriction injury (CCI) (a model of painful peripheral neuropathy), Remacle et al. [97] observed an initial increase in nerve MMP9 expression at day 1 post-CCI, which was then overridden more than 100-fold at day 28 post-CCI. These data confirm the earlier observations and demonstrate the drastic increase in MMP9 expression and activity in the late-phase painful neuropathy. Moreover, this study showed that the high level of MMP9 expression in late-phase nerve injury was accompanied by the reduction in TIMP-1 level, indicating that the excess MMP9 activity was not balanced by its endogenous inhibitor TIMP-1, specifically during the late-phase. MMP9 has been proposed to play a role in pain sustenance and resolution through the control of nerve regeneration, demyelination/remyelination processes, and ion channel functioning [97].

## 10. Conclusions

The failure in the regeneration of injured peripheral nerves can lead to motor and sensory deficits, ending with paralysis and chronic pain. Axonal regeneration is greatly supported by Schwann cells that after injury de-differentiate and guide axons to their original target tissue. In this review, we summarize the most recent findings describing how the pro-regenerative capacities of Schwann cells can be modulated by thrombin acting through its main receptor PAR1. After peripheral nerve injury, the activity of thrombin, the key mediator of coagulation, is known to rapidly rise partially coming from blood extravasation, but also from being locally generated. Together with thrombin, many other proteases are also released at sites of nerve injury and some of them (FXa, FVIIa, APC, plasmin, and some MMPs) can cut and signal through PAR1.

Overall, the results reported in this review indicate that the stimulation of glial PAR1 needs to be finely tuned in order to ensure optimal nerve regeneration. In fact, an excessive activation of the PAR1/thrombin axis during peripheral nerve injury could limit the trophic support of Schwann cells to axonal regrowth. In this view, a pharmacologic inhibition of PAR1 activation could possibly restore the Schwann cell pro-regenerative functions and favor nerve functional recovery. Future studies are needed to clarify the mutual complex regulation of the different PAR1-activating proteases in modulating peripheral nerve regeneration.

## Figures and Tables

**Table 1:** Potential PAR1 activating proteases in peripheral nerve injury.

PAR1 Activating Protease	SC Cultures	Peripheral Nerve	Cleavage Site
Thrombin	Low levels, increased release of neurotrophic factors [13]	Low levels, enhanced regeneration [10]	Canonical
	High levels, decreased SC neurotrophic activity [80]	High levels, reduced regeneration [11]	
FXa	Increased release of thrombin in a Schwannoma cell line [9]	Inhibition of FXa restores motor function after injury [9]	Canonical
FVIIa	Expressed in a Schwannoma cell line [9]	Expressed at the nodes of Ranvier [9]	Canonical
APC/EPCR	EPCR expression in a Schwannoma cell line [7]	EPCR increased expression after crush injury [7]	Canonical and noncanonical
Plasmin	Increase SC migration and wrapping of nerve fibers [85]	tPA and uPA promote nerve regeneration after injury [86,87]	Canonical and noncanonical
MMPs	Inhibit SC proliferation [88]	Inhibit nerve regeneration [89]	Canonical and noncanonical
MMP2	Stimulation of SC migration [90] Enhancement of myelination in SC/DRG co-cultures [91]		Noncanonical
MMP3	Inhibition of SC proliferation [92]	In MMP3 KO mice, NMJ are preserved [93]	Noncanonical
MMP8		Localized at growth cones in nerve fibers [94]	Canonical
MMP9	Inhibition of proliferation and trophic activity of SCs [95,96] Stimulation of SC migration [90]	Upregulated after nerve injury [97]	Canonical and noncanonical

## References

[1] Burnett M.G., Zager E.L. (2004). Pathophysiology of Peripheral Nerve Injury: A Brief Review. Neurosurg. Focus.

[2] Grinsell D., Keating C.P. (2014). Peripheral Nerve Reconstruction after Injury: A Review of Clinical and Experimental Therapies. BioMed Res. Int..

[3] Siemionow M., Brzezicki G. (2009). Chapter 8 Current Techniques and Concepts in Peripheral Nerve Repair. International Review of Neurobiology.

[4] Johnson E.O., Charchanti A., Soucacos P.N. (2008). Nerve Repair: Experimental and Clinical Evaluation of Neurotrophic Factors in Peripheral Nerve Regeneration. Injury.

[5] Jessen K.R., Mirsky R. (2019). The Success and Failure of the Schwann Cell Response to Nerve Injury. Front. Cell. Neurosci..

[6] Schmalbruch H. (1986). Fiber Composition of the Rat Sciatic Nerve. Anat. Rec..

[7] Gera O., Shavit-Stein E., Bushi D., Harnof S., Shimon M.B., Weiss R., Golderman V., Dori A., Maggio N., Finegold K. (2016). Thrombin and Protein C Pathway in Peripheral Nerve Schwann Cells. Neuroscience.

[8] Henderson J.M., Stein S.F., Kutner M., Wiles M.-B., Ansley J.D., Rudman D. (1980). Analysis of Twenty-Three Plasma Proteins in Ascites The Depletion of Fibrinogen and Plasminogen. Ann. Surg..

[9] Gera O., Bushi D., Shimon M.B., Artan-Furman A., Harnof S., Maggio N., Dori A., Chapman J., Shavit-Stein E. (2018). Local Regulation of Thrombin Activity by Factor Xa in Peripheral Nerve Schwann Cells. Neuroscience.

[10] Balezina O.P., Gerasimenko N.Y., Dugina T.N., Strukova S.M. (2005). Study of Neurotrophic Activity of Thrombin on the Model of Regenerating Mouse Nerve. Bull. Exp. Biol. Med..

[11] Lee P., Spector J.G., Derby A., Roufa D.G. (1998). Effects of Thrombin and Protease Nexin-1 on Peripheral Nerve Regeneration. Ann. Otol. Rhinol. Laryngol..

[12] Shavit E., Beilin O., Korczyn A.D., Sylantiev C., Aronovich R., Drory V.E., Gurwitz D., Horresh I., Bar-Shavit R., Peles E. (2008). Thrombin Receptor PAR-1 on Myelin at the Node of Ranvier: A New Anatomy and Physiology of Conduction Block. Brain.

[13] Pompili E., Fabrizi C., Somma F., Correani V., Maras B., Schininà M.E., Ciraci V., Artico M., Fornai F., Fumagalli L. (2017). PAR1 Activation Affects the Neurotrophic Properties of Schwann Cells. Mol. Cell. Neurosci..

[14] Kanno H., Pressman Y., Moody A., Berg R., Muir E.M., Rogers J.H., Ozawa H., Itoi E., Pearse D.D., Bunge M.B. (2014). Combination of Engineered Schwann Cell Grafts to Secrete Neurotrophin and Chondroitinase Promotes Axonal Regeneration and Locomotion after Spinal Cord Injury. J. Neurosci..

[15] Babetto E., Wong K.M., Beirowski B. (2020). A Glycolytic Shift in Schwann Cells Supports Injured Axons. Nat. Neurosci..

[16] Whalley K. (2014). Glia: Schwann Cells Provide Life Support for Axons. Nat. Rev. Neurosci..

[17] Cohen C.C.H., Popovic M.A., Klooster J., Weil M.-T., Möbius W., Nave K.-A., Kole M.H.P. (2020). Saltatory Conduction along Myelinated Axons Involves a Periaxonal Nanocircuit. Cell.

[18] Lim B.C., Rasband M.N. (2020). Saltatory Conduction: Jumping to New Conclusions. Curr. Biol..

[19] Hursh J.B. (1939). Conduction velocity and diameter of nerve fibers. Am. J. Physiol. Leg. Content.

[20] Rushton W.A.H. (1951). A Theory of the Effects of Fibre Size in Medullated Nerve. J. Physiol..

[21] Nave K.-A., Werner H.B. (2021). Ensheathment and Myelination of Axons: Evolution of Glial Functions. Annu. Rev. Neurosci..

[22] Le Douarin N.M., Smith J. (1988). Development of the Peripheral Nervous System from the Neural Crest. Annu. Rev. Cell Biol..

[23] Le Douarin N., Dulac C., Dupin E., Cameron-Curry P. (1991). Glial Cell Lineages in the Neural Crest. Glia.

[24] Buchstaller J., Sommer L., Bodmer M., Hoffmann R., Suter U., Mantei N. (2004). Efficient Isolation and Gene Expression Profiling of Small Numbers of Neural Crest Stem Cells and Developing Schwann Cells. J. Neurosci..

[25] Webster H.D. (1971). The Geometry of Peripheral Myelin Sheaths during Their Formation and Growth in Rat Sciatic Nerves. J. Cell Biol..

[26] Webster H.D., Martin R., O’Connell M.F. (1973). The Relationships between Interphase Schwann Cells and Axons before Myelination: A Quantitative Electron Microscopic Study. Dev. Biol..

[27] Michailov G.V., Sereda M.W., Brinkmann B.G., Fischer T.M., Haug B., Birchmeier C., Role L., Lai C., Schwab M.H., Nave K.-A. (2004). Axonal Neuregulin-1 Regulates Myelin Sheath Thickness. Science.

[28] Bosse F., Hasenpusch-Theil K., Küry P., Müller H.W. (2006). Gene Expression Profiling Reveals That Peripheral Nerve Regeneration Is a Consequence of Both Novel Injury-Dependent and Reactivated Developmental Processes. J. Neurochem..

[29] Gomez-Sanchez J.A., Pilch K.S., van der Lans M., Fazal S.V., Benito C., Wagstaff L.J., Mirsky R., Jessen K.R. (2017). After Nerve Injury, Lineage Tracing Shows That Myelin and Remak Schwann Cells Elongate Extensively and Branch to Form Repair Schwann Cells, Which Shorten Radically on Remyelination. J. Neurosci..

[30] Perry V.H., Tsao J.W., Fearn S., Brown M.C. (1995). Radiation-Induced Reductions in Macrophage Recruitment Have Only Slight Effects on Myelin Degeneration in Sectioned Peripheral Nerves of Mice. Eur. J. Neurosci..

[31] Balakrishnan A., Belfiore L., Chu T.-H., Fleming T., Midha R., Biernaskie J., Schuurmans C. (2020). Insights Into the Role and Potential of Schwann Cells for Peripheral Nerve Repair From Studies of Development and Injury. Front. Mol. Neurosci..

[32] Jessen K.R., Mirsky R. (2016). The Repair Schwann Cell and Its Function in Regenerating Nerves. J. Physiol..

[33] Wagstaff L.J., Gomez-Sanchez J.A., Fazal S.V., Otto G.W., Kilpatrick A.M., Michael K., Wong L.Y., Ma K.H., Turmaine M., Svaren J. (2021). Failures of Nerve Regeneration Caused by Aging or Chronic Denervation Are Rescued by Restoring Schwann Cell C-Jun. eLife.

[34] Gomez-Sanchez J.A., Carty L., Iruarrizaga-Lejarreta M., Palomo-Irigoyen M., Varela-Rey M., Griffith M., Hantke J., Macias-Camara N., Azkargorta M., Aurrekoetxea I. (2015). Schwann Cell Autophagy, Myelinophagy, Initiates Myelin Clearance from Injured Nerves. J. Cell Biol..

[35] Price M., Badaut J., Thevenet J., Hirt L. (2010). Activation of C-Jun in the Nuclei of Neurons of the CA-1 in Thrombin Preconditioning Occurs via PAR-1. J. Neurosci. Res..

[36] Bolívar S., Navarro X., Udina E. (2020). Schwann Cell Role in Selectivity of Nerve Regeneration. Cells.

[37] Simón D., Martín-Bermejo M.J., Gallego-Hernández M.T., Pastrana E., García-Escudero V., García-Gómez A., Lim F., Díaz-Nido J., Avila J., Moreno-Flores M.T. (2011). Expression of Plasminogen Activator Inhibitor-1 by Olfactory Ensheathing Glia Promotes Axonal Regeneration. Glia.

[38] Pompili E., Fabrizi C., Fornai F., Fumagalli L. (2019). Role of the Protease-Activated Receptor 1 in Regulating the Function of Glial Cells within Central and Peripheral Nervous System. J. Neural Transm..

[39] Yoon H., Choi C.-I., Triplet E.M., Langley M.R., Kleppe L.S., Kim H.N., Simon W.L., Scarisbrick I.A. (2020). Blocking the Thrombin Receptor Promotes Repair of Demyelinated Lesions in the Adult Brain. J. Neurosci..

[40] Pompili E., Fabrizi C. (2021). Thrombin in Peripheral Nerves: Friend or Foe?. Neural Regen. Res..

[41] Vu T.K., Hung D.T., Wheaton V.I., Coughlin S.R. (1991). Molecular Cloning of a Functional Thrombin Receptor Reveals a Novel Proteolytic Mechanism of Receptor Activation. Cell.

[42] Nystedt S., Emilsson K., Wahlestedt C., Sundelin J. (1994). Molecular Cloning of a Potential Proteinase Activated Receptor. Proc. Natl. Acad. Sci. USA.

[43] Ishihara H., Connolly A.J., Zeng D., Kahn M.L., Zheng Y.W., Timmons C., Tram T., Coughlin S.R. (1997). Protease-Activated Receptor 3 Is a Second Thrombin Receptor in Humans. Nature.

[44] Xu W.F., Andersen H., Whitmore T.E., Presnell S.R., Yee D.P., Ching A., Gilbert T., Davie E.W., Foster D.C. (1998). Cloning and Characterization of Human Protease-Activated Receptor 4. Proc. Natl. Acad. Sci. USA.

[45] Blanc-Brude O.P., Archer F., Leoni P., Derian C., Bolsover S., Laurent G.J., Chambers R.C. (2005). Factor Xa Stimulates Fibroblast Procollagen Production, Proliferation, and Calcium Signaling via PAR1 Activation. Exp. Cell Res..

[46] Schuepbach R.A., Riewald M. (2010). Coagulation Factor Xa Cleaves Protease-Activated Receptor-1 and Mediates Signaling Dependent on Binding to the Endothelial Protein C Receptor. J. Thromb. Haemost..

[47] Sen P., Gopalakrishnan R., Kothari H., Keshava S., Clark C.A., Esmon C.T., Pendurthi U.R., Rao L.V.M. (2011). Factor VIIa Bound to Endothelial Cell Protein C Receptor Activates Protease Activated Receptor-1 and Mediates Cell Signaling and Barrier Protection. Blood.

[48] Kuliopulos A., Covic L., Seeley S.K., Sheridan P.J., Helin J., Costello C.E. (1999). Plasmin Desensitization of the PAR1 Thrombin Receptor: Kinetics, Sites of Truncation, and Implications for Thrombolytic Therapy. Biochemistry.

[49] Sebastiano M., Momi S., Falcinelli E., Bury L., Hoylaerts M.F., Gresele P. (2017). A Novel Mechanism Regulating Human Platelet Activation by MMP-2-Mediated PAR1 Biased Signaling. Blood.

[50] Lee S.E., Kim J.-M., Jeong S.K., Jeon J.E., Yoon H.-J., Jeong M.-K., Lee S.H. (2010). Protease-Activated Receptor-2 Mediates the Expression of Inflammatory Cytokines, Antimicrobial Peptides, and Matrix Metalloproteinases in Keratinocytes in Response to Propionibacterium Acnes. Arch. Derm. Res..

[51] Nakayama T., Hirano K., Shintani Y., Nishimura J., Nakatsuka A., Kuga H., Takahashi S., Kanaide H. (2003). Unproductive Cleavage and the Inactivation of Protease-Activated Receptor-1 by Trypsin in Vascular Endothelial Cells. Br. J. Pharm..

[52] Suidan H.S., Bouvier J., Schaerer E., Stone S.R., Monard D., Tschopp J. (1994). Granzyme A Released upon Stimulation of Cytotoxic T Lymphocytes Activates the Thrombin Receptor on Neuronal Cells and Astrocytes. Proc. Natl. Acad. Sci. USA.

[53] Lee P.R., Johnson T.P., Gnanapavan S., Giovannoni G., Wang T., Steiner J.P., Medynets M., Vaal M.J., Gartner V., Nath A. (2017). Protease-Activated Receptor-1 Activation by Granzyme B Causes Neurotoxicity That Is Augmented by Interleukin-1β. J. Neuroinflammat..

[54] Wang T., Lee M.-H., Choi E., Pardo-Villamizar C.A., Lee S.B., Yang I.H., Calabresi P.A., Nath A. (2012). Granzyme B-Induced Neurotoxicity Is Mediated via Activation of PAR-1 Receptor and Kv1.3 Channel. PLoS ONE.

[55] Wilson T.J., Nannuru K.C., Singh R.K. (2009). Cathepsin G Recruits Osteoclast Precursors via Proteolytic Activation of Protease-Activated Receptor-1. Cancer Res..

[56] Nystedt S., Emilsson K., Larsson A.K., Strömbeck B., Sundelin J. (1995). Molecular Cloning and Functional Expression of the Gene Encoding the Human Proteinase-Activated Receptor 2. Eur. J. Biochem..

[57] Berger P., Perng D.W., Thabrew H., Compton S.J., Cairns J.A., McEuen A.R., Marthan R., Tunon De Lara J.M., Walls A.F. (2001). Tryptase and Agonists of PAR-2 Induce the Proliferation of Human Airway Smooth Muscle Cells. J. Appl. Physiol..

[58] Rothmeier A.S., Liu E., Chakrabarty S., Disse J., Mueller B.M., Østergaard H., Ruf W. (2018). Identification of the Integrin-Binding Site on Coagulation Factor VIIa Required for Proangiogenic PAR2 Signaling. Blood.

[59] Morris D.R., Ding Y., Ricks T.K., Gullapalli A., Wolfe B.L., Trejo J. (2006). Protease-Activated Receptor-2 Is Essential for Factor VIIa and Xa-Induced Signaling, Migration, and Invasion of Breast Cancer Cells. Cancer Res..

[60] Dömötör E., Bartha K., Machovich R., Adam-Vizi V. (2002). Protease-Activated Receptor-2 (PAR-2) in Brain Microvascular Endothelium and Its Regulation by Plasmin and Elastase. J. Neurochem..

[61] Mihara K., Ramachandran R., Saifeddine M., Hansen K.K., Renaux B., Polley D., Gibson S., Vanderboor C., Hollenberg M.D. (2016). Thrombin-Mediated Direct Activation of Proteinase-Activated Receptor-2: Another Target for Thrombin Signaling. Mol. Pharm..

[62] Oikonomopoulou K., Hansen K.K., Saifeddine M., Tea I., Blaber M., Blaber S.I., Scarisbrick I., Andrade-Gordon P., Cottrell G.S., Bunnett N.W. (2006). Proteinase-Activated Receptors, Targets for Kallikrein Signaling. J. Biol. Chem..

[63] Kaufmann R., Schulze B., Krause G., Mayr L.M., Settmacher U., Henklein P. (2005). Proteinase-Activated Receptors (PARs)—The PAR3 Neo-N-Terminal Peptide TFRGAP Interacts with PAR1. Regul. Pept..

[64] Jacques S.L., Kuliopulos A. (2003). Protease-Activated Receptor-4 Uses Dual Prolines and an Anionic Retention Motif for Thrombin Recognition and Cleavage. Biochem. J..

[65] Ma L., Hollenberg M.D., Wallace J.L. (2001). Thrombin-Induced Platelet Endostatin Release Is Blocked by a Proteinase Activated Receptor-4 (PAR4) Antagonist. Br. J. Pharm..

[66] Gomides L.F., Duarte I.D., Ferreira R.G., Perez A.C., Francischi J.N., Klein A. (2012). Proteinase-Activated Receptor-4 Plays a Major Role in the Recruitment of Neutrophils Induced by Trypsin or Carrageenan during Pleurisy in Mice. Pharmacology.

[67] Sambrano G.R., Huang W., Faruqi T., Mahrus S., Craik C., Coughlin S.R. (2000). Cathepsin G Activates Protease-Activated Receptor-4 in Human Platelets. J. Biol. Chem..

[68] Coughlin S.R. (1999). How the Protease Thrombin Talks to Cells. Proc. Natl. Acad. Sci. USA.

[69] Adams M.N., Ramachandran R., Yau M.-K., Suen J.Y., Fairlie D.P., Hollenberg M.D., Hooper J.D. (2011). Structure, Function and Pathophysiology of Protease Activated Receptors. Pharmacol. Ther..

[70] Wolfe B.L., Trejo J. (2007). Clathrin-Dependent Mechanisms of G Protein-Coupled Receptor Endocytosis. Traffic.

[71] Trejo J., Hammes S.R., Coughlin S.R. (1998). Termination of Signaling by Protease-Activated Receptor-1 Is Linked to Lysosomal Sorting. Proc. Natl. Acad. Sci. USA.

[72] Han X., Nieman M.T. (2020). The Domino Effect Triggered by the Tethered Ligand of the Protease Activated Receptors. Thromb. Res..

[73] Chandrabalan A., Ramachandran R. (2021). Molecular Mechanisms Regulating Proteinase-Activated Receptors (PARs). FEBS J..

[74] Suo Z., Wu M., Ameenuddin S., Anderson H.E., Zoloty J.E., Citron B.A., Andrade-Gordon P., Festoff B.W. (2002). Participation of Protease-Activated Receptor-1 in Thrombin-Induced Microglial Activation. J. Neurochem..

[75] Naldini A., Bernini C., Pucci A., Carraro F. (2005). Thrombin-Mediated IL-10 up-Regulation Involves Protease-Activated Receptor (PAR)-1 Expression in Human Mononuclear Leukocytes. J. Leukoc. Biol..

[76] Niessen F., Schaffner F., Furlan-Freguia C., Pawlinski R., Bhattacharjee G., Chun J., Derian C.K., Andrade-Gordon P., Rosen H., Ruf W. (2008). Dendritic Cell PAR1-S1P3 Signalling Couples Coagulation and Inflammation. Nature.

[77] Fabrizi C., Pompili E., Panetta B., Nori S.L., Fumagalli L. (2009). Protease-Activated Receptor-1 Regulates Cytokine Production and Induces the Suppressor of Cytokine Signaling-3 in Microglia. Int. J. Mol. Med..

[78] Zhao P., Metcalf M., Bunnett N.W. (2014). Biased Signaling of Protease-Activated Receptors. Front. Endocrinol..

[79] Lin H., Liu A.P., Smith T.H., Trejo J. (2013). Cofactoring and Dimerization of Proteinase-Activated Receptors. Pharm. Rev..

[80] Pompili E., Ciraci V., Leone S., De Franchis V., Familiari P., Matassa R., Familiari G., Tata A.M., Fumagalli L., Fabrizi C. (2020). Thrombin Regulates the Ability of Schwann Cells to Support Neuritogenesis and to Maintain the Integrity of the Nodes of Ranvier. Eur. J. Histochem..

[81] Smirnova I.V., Ma J.Y., Citron B.A., Ratzlaff K.T., Gregory E.J., Akaaboune M., Festoff B.W. (1996). Neural Thrombin and Protease Nexin I Kinetics after Murine Peripheral Nerve Injury. J. Neurochem..

[82] Festoff B.W., Citron B.A. (2019). Thrombin and the Coag-Inflammatory Nexus in Neurotrauma, ALS, and Other Neurodegenerative Disorders. Front. Neurol..

[83] Court F.A., Sherman D.L., Pratt T., Garry E.M., Ribchester R.R., Cottrell D.F., Fleetwood-Walker S.M., Brophy P.J. (2004). Restricted Growth of Schwann Cells Lacking Cajal Bands Slows Conduction in Myelinated Nerves. Nature.

[84] Burda J.E., Radulovic M., Yoon H., Scarisbrick I.A. (2013). Critical Role for PAR1 in Kallikrein 6-Mediated Oligodendrogliopathy. Glia.

[85] Kalderon N. (1979). Migration of Schwann Cells and Wrapping of Neurites in Vitro: A Function of Protease Activity (Plasmin) in the Growth Medium. Proc. Natl. Acad. Sci. USA.

[86] Sajadi E., Aliaghaei A., Farahni R.M., Rashidiani-Rashidabadi A., Raoofi A., Sadeghi Y., Bagheri M., Ilkhani S., Abdollahifar M.-A. (2021). Tissue Plasminogen Activator Loaded PCL Nanofibrous Scaffold Promoted Nerve Regeneration After Sciatic Nerve Transection in Male Rats. Neurotox. Res..

[87] Klimovich P.S., Semina E.V., Karagyaur M.N., Rysenkova K.D., Sysoeva V.Y., Mironov N.A., Sagaradze G.D., Az’muko A.A., Popov V.S., Rubina K.A. (2020). Urokinase Receptor Regulates Nerve Regeneration through Its Interaction with A5β1-Integrin. Biomed. Pharm..

[88] Liu H., Kim Y., Chattopadhyay S., Shubayev I., Dolkas J., Shubayev V.I. (2010). Matrix Metalloproteinase Inhibition Enhances the Rate of Nerve Regeneration In Vivo by Promoting Dedifferentiation and Mitosis of Supporting Schwann Cells. J. Neuropathol. Exp. Neurol..

[89] Willis Fox O., Preston R.J.S. (2020). Molecular Basis of Protease-activated Receptor 1 Signaling Diversity. J. Thromb. Haemost..

[90] Muscella A., Vetrugno C., Cossa L.G., Marsigliante S. (2020). TGF-Β1 Activates RSC96 Schwann Cells Migration and Invasion through MMP-2 and MMP-9 Activities. J. Neurochem..

[91] Lehmann H.C., Köhne A., Bernal F., Jangouk P., Meyer Zu Hörste G., Dehmel T., Hartung H.-P., Previtali S.C., Kieseier B.C. (2009). Matrix Metalloproteinase-2 Is Involved in Myelination of Dorsal Root Ganglia Neurons. Glia.

[92] Muir D. (1995). Differences in Proliferation and Invasion by Normal, Transformed and NF1 Schwann Cell Cultures Are Influenced by Matrix Metalloproteinase Expression. Clin. Exp. Metastasis.

[93] Chao T., Frump D., Lin M., Caiozzo V.J., Mozaffar T., Steward O., Gupta R. (2013). Matrix Metalloproteinase 3 Deletion Preserves Denervated Motor Endplates after Traumatic Nerve Injury. Ann. Neurol..

[94] Tominaga M., Tengara S., Kamo A., Ogawa H., Takamori K. (2011). Matrix Metalloproteinase-8 Is Involved in Dermal Nerve Growth: Implications for Possible Application to Pruritus from in Vitro Models. J. Investig. Derm..

[95] Chattopadhyay S., Myers R.R., Janes J., Shubayev V. (2007). Cytokine Regulation of MMP-9 in Peripheral Glia: Implications for Pathological Processes and Pain in Injured Nerve. Brain Behav. Immun..

[96] Shubayev V.I., Angert M., Dolkas J., Campana W.M., Palenscar K., Myers R.R. (2006). TNFalpha-Induced MMP-9 Promotes Macrophage Recruitment into Injured Peripheral Nerve. Mol. Cell Neurosci..

[97] Remacle A.G., Hullugundi S.K., Dolkas J., Angert M., Chernov A.V., Strongin A.Y., Shubayev V.I. (2018). Acute- and Late-Phase Matrix Metalloproteinase (MMP)-9 Activity Is Comparable in Female and Male Rats after Peripheral Nerve Injury. J. Neuroinflammat..

[98] Petzold T., Thienel M., Dannenberg L., Mourikis P., Helten C., Ayhan A., M’Pembele R., Achilles A., Trojovky K., Konsek D. (2020). Rivaroxaban Reduces Arterial Thrombosis by Inhibition of FXa-Driven Platelet Activation via Protease Activated Receptor-1. Circ. Res..

[99] Kondreddy V., Pendurthi U.R., Xu X., Griffin J.H., Rao L.V.M. (2020). FVIIa (Factor VIIa) Induces Biased Cytoprotective Signaling in Mice Through the Cleavage of PAR (Protease-Activated Receptor)-1 at Canonical Arg41 (Arginine41) Site. Arter. Thromb. Vasc. Biol..

[100] Wojtukiewicz M.Z., Hempel D., Sierko E., Tucker S.C., Honn K.V. (2019). Endothelial Protein C Receptor (EPCR), Protease Activated Receptor-1 (PAR-1) and Their Interplay in Cancer Growth and Metastatic Dissemination. Cancers.

[101] Lin Y., Wozniak J.M., Grimsey N.J., Girada S., Patwardhan A., Molinar-Inglis O., Smith T.H., Lapek J.D., Gonzalez D.J., Trejo J. (2020). Phosphoproteomic Analysis of Protease-Activated Receptor-1 Biased Signaling Reveals Unique Modulators of Endothelial Barrier Function. Proc. Natl. Acad. Sci. USA.

[102] Mosnier L.O., Sinha R.K., Burnier L., Bouwens E.A., Griffin J.H. (2012). Biased Agonism of Protease-Activated Receptor 1 by Activated Protein C Caused by Noncanonical Cleavage at Arg46. Blood.

[103] Soh U.J.K., Trejo J. (2011). Activated Protein C Promotes Protease-Activated Receptor-1 Cytoprotective Signaling through β-Arrestin and Dishevelled-2 Scaffolds. Proc. Natl. Acad. Sci. USA.

[104] García-Rocha M., Avila J., Armas-Portela R. (1994). Tissue-Type Plasminogen Activator (TPA) Is the Main Plasminogen Activator Associated with Isolated Rat Nerve Growth Cones. Neurosci. Lett..

[105] Siconolfi L.B., Seeds N.W. (2001). Mice Lacking TPA, UPA, or Plasminogen Genes Showed Delayed Functional Recovery after Sciatic Nerve Crush. J. Neurosci..

[106] Siconolfi L.B., Seeds N.W. (2003). Mice Lacking Tissue Plasminogen Activator and Urokinase Plasminogen Activator Genes Show Attenuated Matrix Metalloproteases Activity after Sciatic Nerve Crush. J. Neurosci. Res..

[107] Hantaï D., Rao J.S., Festoff B.W. (1990). Rapid Neural Regulation of Muscle Urokinase-like Plasminogen Activator as Defined by Nerve Crush. Proc. Natl. Acad. Sci. USA.

[108] Akassoglou K., Kombrinck K.W., Degen J.L., Strickland S. (2000). Tissue Plasminogen Activator-Mediated Fibrinolysis Protects against Axonal Degeneration and Demyelination after Sciatic Nerve Injury. J. Cell Biol..

[109] Nagase H., Visse R., Murphy G. (2006). Structure and Function of Matrix Metalloproteinases and TIMPs. Cardiovasc. Res..

[110] Bonnans C., Chou J., Werb Z. (2014). Remodelling the Extracellular Matrix in Development and Disease. Nat. Rev. Mol. Cell Biol..

[111] Cabral-Pacheco G.A., Garza-Veloz I., Castruita-De la Rosa C., Ramirez-Acuña J.M., Perez-Romero B.A., Guerrero-Rodriguez J.F., Martinez-Avila N., Martinez-Fierro M.L. (2020). The Roles of Matrix Metalloproteinases and Their Inhibitors in Human Diseases. Int. J. Mol. Sci..

[112] Demestre M., Wells G.M., Miller K.M., Smith K.J., Hughes R.A.C., Gearing A.J., Gregson N.A. (2004). Characterisation of Matrix Metalloproteinases and the Effects of a Broad-Spectrum Inhibitor (BB-1101) in Peripheral Nerve Regeneration. Neuroscience.

[113] Han S., Kim D.H., Sung J., Yang H., Park J.W., Youn I. (2019). Electrical Stimulation Accelerates Neurite Regeneration in Axotomized Dorsal Root Ganglion Neurons by Increasing MMP-2 Expression. Biochem. Biophys. Res. Commun..

[114] Ferguson T.A., Muir D. (2000). MMP-2 and MMP-9 Increase the Neurite-Promoting Potential of Schwann Cell Basal Laminae and Are Upregulated in Degenerated Nerve. Mol. Cell Neurosci..

[115] Ali S., Driscoll H.E., Newton V.L., Gardiner N.J. (2014). Matrix Metalloproteinase-2 Is Downregulated in Sciatic Nerve by Streptozotocin Induced Diabetes and/or Treatment with Minocycline: Implications for Nerve Regeneration. Exp. Neurol..

[116] Parks W.C., Wilson C.L., López-Boado Y.S. (2004). Matrix Metalloproteinases as Modulators of Inflammation and Innate Immunity. Nat. Rev. Immunol..

[117] Fang X., Chen J., Wang W., Feng G., Li X., Zhang X., Zhang Y., Zhang J., Xu Z., Tai J. (2020). Matrix Metalloproteinase 9 (MMP9) Level and MMP9 -1562C>T in Patients with Obstructive Sleep Apnea: A Systematic Review and Meta-Analysis of Case-Control Studies. Sleep Med..

